# The Super-Child

**Published:** 1919-08-02

**Authors:** 


### THE SUPER-CHILD.

### Progressive Ideas and a Nation's Simple Duty.

The present is almost necessarily a time of much cry and little wool. From every quarter come proposals and resolutions and schemes, plans of reform, renovation and construction. Those who have been saving their breath during the last four or five years can now hardly hear each other speak. In fact, it is almost time to interdict more words, and proceed forthwith to action. In this respect a good example has been set by some of the leading sanitary authorities of the country. Touring many large areas will be found small organisations—mostly in the form of an "exhibition" with a permanent lecturer—which stop for a day or two at local centres, enlist the help of local sanitary officials, and get on with the great work of educating local public opinion. We refer especially to the child welfare movement. The great truths of sanitary science, dating in this country from the time of de Chaumont and Parkes, have been found to have particular practical value when applied to puericulture, always provided that detailed popular instruction is made easily available to every class of parent. The richer sort can get the needed information from books and from their family medical attendant, but obviously this is not the case with the working-class. The virtues of the wide-open window, of weekly weighing of infants, some elementary physiology of the neo-natal nervous system, intelligently practised, have a very notably beneficial effect upon the health and happiness of the rising generation.

And surely England should be a rich field for such propaganda. No other country is so famed for stock raising; all the breeds of the domesticated animals are cultivated to perfection here; and there seems every probability that the young of the human species should be so too. Where "fanciers" abound it should not be difficult, even in large towns, to get children raised who with their rosy faces and clear skins would rival the appearance of Derby favourites or show poultry or prize cattle. At present, in urban areas at least, this delightful kind of super-child is seen only in the upper middle classes. Except after a summer holiday the artisan's offspring are pretty uniformly pale, lack lustre in hair and eyes, short of bloom and condition, and skinny or puffy in body. If this be thought over-stated, contrast the smaller girls at a paying secondary school with the bigger ones at a council school. In comparison the latter will seem as has been described, and their lack of weight and development will also be noticeable. But given some social amelioration, some improvement in opportunity and resources, work such as we have mentioned, aided by private effort like that of the Invalid Children Association, which is holding a meeting early next month at Manchester, will make the super-child, which is merely what every child should be, a much more widely encountered phenomenon. As yet, in truth, puericulture is altogether inchoate. Seeing what we now know of the infantile origin of disease, it is destined to develop enormously in coming years. Butler's satire of putting a young man upon his trial for the offence of getting consumption may come true this much, that his parents and instructors will have some bad quarters of an hour to experience, if nothing further.

Besides this particular justification there are much wider grounds, of course, upon which to base the rationale of child-welfare work. It is a physical education—it is even the beginnings of a scientific education—which should work a mighty change for the better in the habits of the nation. Adults will seldom alter their insanitary ways, and after all they have a little reason on their side. They have survived the foolish things they have done, and often flourished in face of unhygienic conditions. Those to whom such conditions were really baneful have mostly perished by the way; and the existing adult population represents to some extent the naturally immune. Perhaps, if they were suddenly to change their mode of life, the result might not be altogether a successful one. Anyhow, the progressive stiffening of the mind, which sets in once adolescence is passed removes the likelihood of their doing so.

### The Outlook on Health and Disease.

It is for the sanitary workers to train up the rising generation in better habits, with a better-ordered outlook upon health and disease, and a better conception of the causation of health and disease is the stepping-stone to a better conception of the causation of much else. Hence, in time, a state of public opinion, which would support fewer quacks, patent-medicine vendors, alcohol traders; fewer fortune-tellers, spiritualists, magicians of all kinds; perhaps fewer prostitutes. The golden age will not be reached in a generation, for ill-nature is stronger even than ill-nurture. But all the same the sanitarian is on strong grounds when he claims to be rationally a meliorist. However things were in Peacock's day, the meliorist now in certain directions has plainly the advantage in argument over both the pessimist and the "statu quo-ite." Most particularly is the sanitarian justified when he pays attention, as he seems to be doing now, to the children. At the same time he must deprecate undue expenditure of time and energy on hopeless weaklings, on the one hand, and on cockered up prodigies on the other. The deleterious racial influence which preservation of the first would exert must be allowed to countervail extravagant efforts to protect them. But to rear large masses of children in possession of bounding health and spirits—in the pink, as one says, of condition—is possible enough if progressive ideas rule. And a nation's simple duty, too, some might say. Certainly no other deed could go further than amelioration of the lot of mankind, of which so much has been sung and said in vain.

